# Modulating Sirtuin Biology and Nicotinamide Adenine Diphosphate Metabolism in Cardiovascular Disease—From Bench to Bedside

**DOI:** 10.3389/fphys.2021.755060

**Published:** 2021-10-12

**Authors:** Yu-Jen Wang, Francesco Paneni, Sokrates Stein, Christian M. Matter

**Affiliations:** ^1^Center for Molecular Cardiology, University of Zurich, Schlieren, Switzerland; ^2^Department of Cardiology, University Heart Center, University Hospital Zurich, Zurich, Switzerland; ^3^Department of Research and Education, University Hospital of Zurich, Zurich, Switzerland

**Keywords:** sirtuins, NAD^+^ boosters, cardiovascular diseases, translational studies, caloric restriction

## Abstract

Sirtuins (SIRT1–7) comprise a family of highly conserved deacetylases with distribution in different subcellular compartments. Sirtuins deacetylate target proteins depending on one common substrate, nicotinamide adenine diphosphate (NAD^+^), thus linking their activities to the status of cellular energy metabolism. Sirtuins had been linked to extending life span and confer beneficial effects in a wide array of immune-metabolic and cardiovascular diseases. SIRT1, SIRT3, and SIRT6 have been shown to provide protective effects in various cardiovascular disease models, by decreasing inflammation, improving metabolic profiles or scavenging oxidative stress. Sirtuins may be activated collectively by increasing their co-substrate NAD^+^. By supplementing NAD^+^ precursors, NAD^+^ boosters confer pan-sirtuin activation with protective cardiometabolic effects in the experimental setting: they improve endothelial dysfunction, protect from experimental heart failure, hypertension and decrease progression of liver steatosis. Different precursor molecules were applied ranging from nicotinamide (NAM), nicotinamide mononucleotide (NMN) to nicotinamide riboside (NR). Notably, not all experimental results showed protective effects. Moreover, the results are not as striking in clinical studies as in the controlled experimental setting. Species differences, (lack of) genetic heterogeneity, different metabolic pathways, dosing, administration routes and disease contexts may account for these challenges in clinical translation. At the clinical scale, caloric restriction can reduce the risks of cardiovascular disease and raise NAD^+^ concentration and sirtuin expression. In addition, antidiabetic drugs such as metformin or SGLT2 inhibitors may confer cardiovascular protection, indirectly via sirtuin activation. Overall, additional mechanistic insight and clinical studies are needed to better understand the beneficial effects of sirtuin activation and NAD^+^ boosters from bench to bedside.

## Sirtuins—Protection in Immunometabolic Disease and Aging

Mammalian sirtuins (SIRT1–SIRT7) comprise a group of enzymes, highly conserved from yeasts to mammalian cells, whose homolog is the yeast silent information regulator 2 (Sir2). After uncovering its mechanisms of action involving the cofactor nicotinamide adenine dinucleotide (NAD^+^), it was shown that Sir2 and eukaryotic homologs have dual functions as metabolic sensors and epigenetic regulators (Imai et al., 2000). SIRTs deacetylate histones and key signaling molecules in a NAD^+^-dependent fashion. The enzymes remove the acetyl group from a lysine residue of the target protein and transfer it to NAD^+^. NAD^+^ receives the acetyl group and is cleaved into nicotinamide (NAM) and 2′-*O*-acetyl-ADP-ribose (2′-AADPR) (Sauve et al., 2001; Sauve and Youn, 2012). Distributed in subcellular compartments, sirtuins target proteins in many organelles in the cell to confer protection in metabolic disease, inflammation, and aging. SIRT1 and SIRT2 are confined to the nucleus and the cytosol. SIRT3–5 are localized in mitochondria, while SIRT6 and 7 are predominantly in the nucleus (Houtkooper et al., 2012).

Of all sirtuins, most is known about SIRT1: *Sirt1* overexpression or pharmacological SIRT1 activation in mice has been shown to prevent metabolic and age-related disease including insulin resistance, obesity and liver steatosis (Lagouge et al., 2006). Conversely, loss-of-function (LOF) in *Sirt1*, *Sirt3*, *Sirt6*, and *Sirt7* was reported to increase susceptibility to metabolic and age-related diseases (Finkel et al., 2009). Interestingly, some sirtuins were shown to affect life span: genetic *Sirt1* deficiency reduced survival in the range of days in mice, whereas lack of *Sirt6* and *Sirt7* impaired life span to weeks or months highlighting the critical role of these nuclear sirtuins for mammalian survival (Finkel et al., 2009; Houtkooper et al., 2012).

**FIGURE 1:**
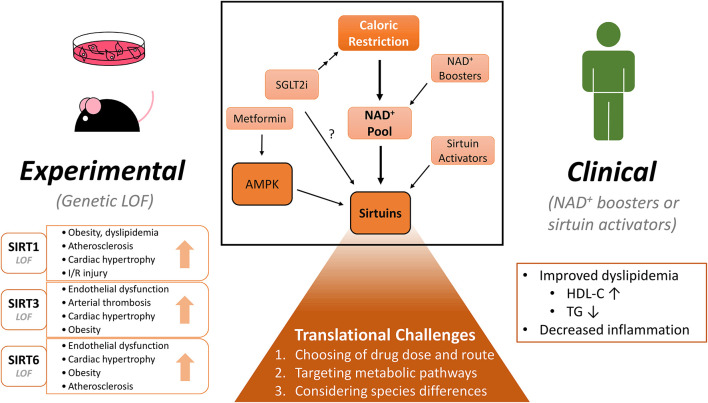
Experimental and clinical benefits of modulating NAD^+^ pool and sirtuins on cardiovascular diseases. CR (Caloric restriction) and NAD^+^ (nicotinamide adenine dinucleotide) boosters raise the NAD^+^ pool within the cell and in turn may activate sirtuins. Similar effects may be induced by pharmacological sirtuin activators. Activation of sirtuins confer numerous beneficial effects: In experimental rodent models, sirtuin isoform loss-of-function studies demonstrate deterioration in cardiometabolic diseases. NAD^+^ boosting also shows some benefits in clinical studies, but more diligent study designs are warranted. When considering clinical translation, several challenges will need to be addressed to determine the clinical potential of these molecules. Lastly, SGLT2 (sodium glucose cotransporter 2) inhibitors show protection in cardiovascular disease. By inducing a state of caloric restriction, they may modulate the NAD^+^ pool and/or sirtuin activity. Many drugs may have unspecific effects, which are not highlighted in this figure. LOF, loss-of-function; I/R, ischemia-reperfusion; NAD^+^, nicotinamide adenine dinucleotide; SGLT2i, sodium-glucose cotransporter 2 inhibitor; HDL-C, high-density lipoprotein cholesterol; TG, triglyceride.

This review focuses on the recent reports of sirtuin-mediated protection in cardiovascular disease (CVD) and pharmaceutical avenues to modulate sirtuin activity. We will discuss the recent progress from the experimental level to human clinical trials and the potential challenges to translate the preclinical data to clinical studies (Figure 1).

## Beneficial Effects of Sirtuins in Cardiovascular Disease

We have previously reviewed the protective effects of sirtuins in metabolic syndromes and CVD at the experimental and clinical level (Winnik et al., 2015) focusing on *Sirt1*, *Sirt3*, *Sirt6*, and *Sirt7*. Here we update the recent findings about these sirtuins and their roles in cardiometabolic diseases.

### Nuclear Sirtuin 1

Caloric restriction upregulates SIRT1 and in turn activates endothelial nitric oxide synthase (eNOS) in mice (Nisoli et al., 2005). SIRT1 can decrease macrophage-based NF-κB activation (Zhang et al., 2008; Stein et al., 2010), thereby diminishing vascular inflammation in the progression of atherosclerosis and arterial thrombosis in mice (Breitenstein et al., 2011). *Sirt1* deficiency (heterozygous knockout) upon high-fat diet induced accumulation of fat mass and increased body weight of mice, which possibly resulted from the decrease in energy expenditure (Xu F. et al., 2016). SIRT1 also showed a protective role in a mouse model of cardiac ischemia-reperfusion: Overexpression of SIRT1 restored cardiac output, whereas heterozygous knockout mice failed to show protection in this model (Hsu et al., 2010; Nadtochiy et al., 2011). Aside from the well-known SIRT1, other sirtuins such as SIRT3 and SIRT6 were identified to exert crucial functions in CVD. We will discuss more about SIRT3 and SIRT6 in the next paragraphs.

### Mitochondrial Sirtuin 3

SIRT3 is located in the mitochondria and regulates acetylation of mitochondrial proteins and confers oxidant stress-relieving effects upon caloric restriction. Such effects are achieved in part through regulation (deacetylation) of superoxide dismutases (SODs) in mitochondria to solve reactive oxygen species (ROS) stress in mice (Qiu et al., 2010; Tao et al., 2010). Recently, a role of endothelial SIRT3 was described in vascular disease where SIRT3 deacetylated SOD2 in knockout mice models (Chen et al., 2017; Traba et al., 2017; Dikalova et al., 2020). The regulation of SIRT3 on mitochondrial ROS scavengers is important under different experimental mice models that stimulate endothelial cells, as evident in angiotensin II-induced hypertension model (Dikalova et al., 2020) or cardiovascular inflammation (Chen et al., 2017; Song et al., 2021). Moreover, SIRT3 conferred protective effects on arterial thrombosis (Gaul et al., 2018), endothelial dysfunction (Winnik et al., 2016) and body weight gain (Winnik et al., 2014) in mice. SIRT3 also played a role in melatonin’s decreased atherosclerosis progression in Apolipoprotein E (*ApoE*)-deficient mice (Ma et al., 2018).

In addition to acting within heart or vascular cells, SIRT3 also regulates the immune cell response. SIRT3 may suppress inflammasome assembly during fasting (Traba et al., 2017), and SIRT3 overexpression reversed the inflammatory cytokine expression in a hypertension model or macrophage stimulation in mice (Liu et al., 2018b; Dikalova et al., 2020). Mechanistically, a recent study demonstrated how SIRT3 regulates cellular energy metabolism: SIRT3 associated with ATP synthase under normal mitochondria membrane potential, but released to activate tricarboxylic acid (TCA) cycle upon lowered mitochondria membrane potential, demonstrated in mice and *in vitro* models (Yang et al., 2016).

Another study showed SIRT3 had protective effect in cardiac hypertrophy through its regulation of nicotinamide mononucleotide adenylyltransferase (NMNAT) 3, which is the enzyme that catalyzes the formation of required substrate NAD^+^. The positive feedback loop needs SIRT3 to activate NMNAT3, producing more NAD^+^ for sirtuins activation, and confers cardioprotection in mice (Yue et al., 2016).

Global genetic deletion of *Sirt3* induced obesity in mice fed a high fat diet, and the phenotype was accredited to modulation of mitochondria-rich, energy-dissipating brown adipose tissue (Gao et al., 2020). These findings shed light on the growing importance of mitochondrial SIRT3 in metabolic control and ROS elimination.

### Nuclear Sirtuin 6

SIRT6 is a nuclear sirtuin, playing a major function in response to DNA damage and suppressing gene expression levels by deacetylating histones of specific sites, such as H3K9, H3K56 (Tasselli et al., 2017). Due to the shortening of life span of the global knockout mice in the range of weeks, many studies applied heterozygote knockout mice or cell-specific deletion.

Several studies reported protective effects of SIRT6 in mice atherosclerotic models (Liu et al., 2016b; Xu S. et al., 2016; Arsiwala et al., 2020). Loss or downregulation of *Sirt6* in *ApoE* knockout mice either had effects on macrophage recruitment or foam cell formation. Our group showed that deficiency of *Sirt6* in bone marrow-derived cells increased atherosclerosis in *ApoE* knockout mice due to increased expression of scavenger receptor macrophage scavenger receptor 1 (MSR1) and foam cell formation (Arsiwala et al., 2020). Moreover, *Sirt6*-depleted endothelial cells expressed increased vascular cell adhesion molecule 1 (VCAM-1) and intercellular adhesion molecule 1 (ICAM-1), which may account for inflammatory cell recruitment in atherosclerotic disease (Liu et al., 2016b; Xu S. et al., 2016). Human samples also showed lower SIRT6 expression in plaques as compared with normal aorta samples (Grootaert et al., 2021). In human vascular smooth muscle cells, SIRT6 suppresses the increase in age-induced inflammatory cytokines (Grootaert et al., 2021). Moreover, endothelial cell-specific deletion of *Sirt6* dampened endothelial-dependent relaxation of blood vessel in mice, suggesting a role of *Sirt6* in endothelial dysfunction (Xu S. et al., 2016). Moreover, it provides beneficial effects in cardiac hypertrophy by suppressing insulin-like growth factor (IGF)-Akt signaling in mouse hearts (Sundaresan et al., 2012).

SIRT6 also protected from diet-induced obesity: systemic overexpression of *Sirt6* decreased fat pads in high-fat diet-treated mice (Kanfi et al., 2010). Later, specific knockout experiments showed that SIRT6 regulated energy homeostasis by modulating adipose tissue lipase expression and activity (Kuang et al., 2017) or regulating appetite to affect food intake of mice (Tang et al., 2020).

Interestingly, a recent paper unveiled that *Sirt6* and *Sirt3* can regulate each other’s expression levels using a diabetic, insulin-resistant mice model (Kanwal et al., 2019). All of the above showed that SIRT3 and SIRT6 conferred protective effects in cardiometabolic disease and their active crosstalk may pave the way for novel therapeutic approaches modulating this important axis in the vasculature and the heart.

## Nicotinamide Adenine Diphosphate Metabolic Pathways

Sirtuins play a huge role in CVD disease and other metabolic diseases as discussed in above sections, and all sirtuins work with NAD^+^. The metabolite is essential for sirtuin activity and exists in oxidized (NAD^+^) and reduced (NADH) form and is a key factor for many redox reactions in the mitochondrial energy chain and posttranslational modifications. Of note, the NAD^+^/NADH ratio reflects the energy state of the cell. NAD^+^ levels are high upon caloric restriction and exercise. NAD^+^ levels are low upon caloric excess, high fat diet, in obesity and type 2 diabetes, DNA damage, cancer, and aging (Mouchiroud et al., 2013).

The availability of NAD^+^ depends on a *de novo* synthesis and a *salvage* pathway. The *de novo* pathway synthesizes NAAD (nicotinic acid adenine dinucleotide) in a two-step mechanism from niacin or a multi-step mechanism from all the way back to tryptophan. NAAD (carboxylate form) can then be converted into NAD^+^ (amide form) by NAD^+^ synthetase. On the other hand, the *salvage* pathway replenishes NAD^+^ from NMN (nicotinamide mononucleotide) (Nakagawa and Guarente, 2014).

NMN can be derived from the phosphorylation of nicotinamide riboside (NR), or from the condensation of ribose-5-phosphate with nicotinamide (NAM) (Nakagawa and Guarente, 2014). Daily requirements for NAD^+^ biosynthesis can be met with consumption of less than 20 mg of niacin, while almost every food can provide NAD^+^ or other “niacin equivalents” (Bogan and Brenner, 2008). It should be noted that not all cell types or tissues can fully utilize different forms of NAD^+^ precursors through the two pathways. Corresponding enzymes have to be expressed to use different precursors (Bogan and Brenner, 2008). As NAD^+^ or NADH cannot diffuse through cell membranes, NAD^+^ availability even differs in the subcellular compartments. Thus, the cellular NAD^+^ pool is regulated in a compartment and tissue-specific fashion (Canto et al., 2015; Ryu et al., 2018).

Supplementation of NAD^+^ using NMN (Yoshino et al., 2011) or NR (Canto et al., 2012) restores NAD^+^ pools and activates SIRT1 (Gomes et al., 2013) and possibly other sirtuins. Increasing NAD^+^ levels improved mitochondrial function under stress and protected from diet-induced obesity and functional decline during aging. Thus, NAD^+^ levels, linking cellular metabolism to changes in signaling and transcriptional events, may respond dynamically over a broad range of physiological conditions and spectrum of diseases (Imai and Guarente, 2014).

Taken together, these findings make pharmacological interventions that increase NAD^+^ levels an attractive therapeutic concept.

## Pharmacologically Increasing Sirtuin Activity in Preclinical Studies

### Sirtuin Activating Compounds

Before we look at pan-sirtuin activators, efforts were made to activate specific sirtuins. While isoform-specific sirtuin activators may ultimately prove useful, such activators are difficult to design. Major attention was attributed to SIRT1-activating compounds (STAC), but the small molecule STACs did not perform well in clinical trials. SRT2104 provided some lipid lowering effects, but blood glucose lowering was either absent or not significant. Anti-inflammatory effects of SRT2104 were found in patients with ulcerative colitis or psoriasis (Krueger et al., 2015; Sands et al., 2016). The results were not as promising as expected and assigned to variable pharmacokinetics upon oral administration of the compound (Dai et al., 2018).

While some STACs exhibited SIRT1-activating ability in animal models, few passed the clinical stress test. SRT3025 showed its modulation of liver PCSK9 to affect lipid profile and confer atheroprotection in mice (Miranda et al., 2015), but its development as clinical drug was stopped. Another STAC, SRT1720 also improved cardiomyocyte function using a mouse model of aging (Zhang et al., 2014). E1231, another STAC, showed promising effects in atherosclerotic *ApoE* knockout mice. E1231 decreases LDL-cholesterol, probably through upregulation of *Abca1* in macrophages and SIRT1-dependent deacetylation of LXRα (Feng et al., 2018). Another interesting STAC E6155, possesses the ability to regulate glucose metabolism in diabetic mice model. E6155 also is showed to directly bind to SIRT1 and it improves blood glucose tolerance and insulin resistance through stimulating glucose uptake in liver and muscle cells, with elevated adiponectin and AMPK (adenosine monophosphate-activated protein kinase) activation of diabetic mice (Liu et al., 2018a).

### Nicotinamide Adenine Diphosphate Boosters

Many studies identified multiple sirtuins including SIRT1, SIRT3, and SIRT6 to provide beneficial effects in CVD and energy metabolism. In addition, activating selectively SIRT1 had shown that not every small compound activator could recapitulate the wide array of SIRT1 activities, let alone all the combined benefits of activating all 7 sirtuins.

Therefore, boosting their common co-substrate NAD^+^ appears a straightforward and effective solution for pan-sirtuin activation. NAD^+^ content can be boosted by direct administration of NAD^+^ precursors (Canto et al., 2015) such as NAM (Yang et al., 2014; Mitchell et al., 2018), NMN (Lee et al., 2016; Tarantini et al., 2019), or NR (Canto et al., 2012; Ratajczak et al., 2016). NAD^+^ boosting showed recently protective effects in many aspects (Kang et al., 2020; Katsyuba et al., 2020). In a recent study, exogenous NAD^+^ application reduced infarction size in an ischemia/reperfusion rat model by regulating autophagy pathways (Zhang et al., 2020). Nevertheless, the major strategies comprise targeting the salvage pathway of NAD^+^ synthesis in the cells by supplementing NMN or NR.

#### Nicotinamide Mononucleotide

NMN supplementation in mice has been found to increase the NAD^+^ pool in mouse aortae. This boosting improved endothelial dysfunction in aged mice (De Picciotto et al., 2016; Tarantini et al., 2019). NMN supplementation as well showed protection in experimental mice models of both heart failure with reduced (HFrEF) and preserved (HFpEF) ejection fraction (Lee et al., 2016).

NMN supplementation also reduced size of myocardial infarction in a mouse ischemia-reperfusion model. Supplementing NMN reduced Forkhead Box O1 (FOXO1) acetylation (as readout of SIRT1 activity) and the protective effects vanished in *Sirt1*-deficient mice (Yamamoto et al., 2014). Another group demonstrated NMN-induced activation of mitochondrial SIRT3 by acetylation of mitochondrial proteins and improving energy metabolism of mouse cardiomyocytes (Nadtochiy et al., 2018). Other than *Sirt1* and *Sirt3*, the effects of NMN supplementation on endothelial cells may be mediated by expression of *Sirt2* in cultured endothelial cells, as after knocking down both *Sirt1* and *Sirt2*, mitochondrial respiration remained decreased (Tarantini et al., 2019). This showed the advantageous effects of activating more than one sirtuin upon NMN supplementation.

#### Nicotinamide Riboside

Another promising NAD^+^ booster candidate is NR. NR fueled the NAD^+^ pool and exerted protective effects in experimental mouse models of HFrEF and HFpEF (Diguet et al., 2018; Tong et al., 2021). NR, whether given in a preventive or in a treatment context, conferred beneficial effects on liver steatosis by upregulating *Sirt1* expression and replenishing NAD^+^ concentration (Gariani et al., 2016). Moreover, restoration of NAD^+^ levels by NAM improved diastolic dysfunction in mouse and rat models of aging, hypertension or cardiometabolic syndrome (Abdellatif et al., 2021) by NAM-induced improvement of myocardial energetics, cardiomyocyte passive stiffness and calcium-dependent active relaxation.

However, there remains some research which does not provide as positive messages. Although Canto et al. (2012) showed convincing results where NR supplementation induced anti-obesity effects in mice, other studies could not reproduce this phenotype. Other groups investigated supplementation of NR in a similar “mildly obesogenic diet” or high-fat diet in mice. These authors did not find body weight changes, even upon high dosages of NR. They also showed increased liver steatosis, impaired blood glucose control in high NR groups (Shi et al., 2017, 2019; Cartwright et al., 2021).

In spite of these negative findings, many researchers still concluded that NR applied experimentally in a controlled dosage (30 mg/kg BW) is beneficial. It may improve metabolic adaptiveness that is better than in a low-supplemented (5 mg/kg BW) or over-supplemented group (900 mg/kg BW) (Shi et al., 2017). Notably, 900 mg/kg BW group showed decreased inflammation and higher anti-oxidant gene expression in the adipose tissue of mice (Shi et al., 2017). The difference of this compared to the previous study (Canto et al., 2012) may result from the genetic background of the experimental mice used that interfered with NAD^+^ generation from its precursors.

At the clinical level, high dietary intake of naturally occurring NAD^+^ precursors was associated with lower blood pressure and reduced risk of cardiac mortality in a long-term human cohort study (Abdellatif et al., 2021); of note, this study was a retrospective analysis subject to confounding, not a randomized controlled trial. These recent findings suggest that NAD^+^ precursors might be leveraged for the treatment of diastolic dysfunction and HFpEF in humans. The relevance of these results is amplified by the lack of effective therapies to treat HFpEF and the dismal prognosis associated with this condition (Pfeffer et al., 2019).

To sum up, NAD^+^ boosting, whether through NMN or NR supplementation, showed some benefits in different animal models of cardiometabolic diseases. These effects were accredited to SIRT1 and/or SIRT3 activation using knockout or knockdown strategies, but we cannot rule out that the raised NAD^+^ pool activated other sirtuins or other sirtuin-independent processes within the cells. Of note, drug dosage and genetic background may alter the experimental phenotypes. We summarize these experimental findings in Table 1.

**TABLE 1:** Pharmacological intervention of sirtuin activity and their effects on cardiovascular risk factors and diseases.

Target	Compound	Effects	References
**Experimental**			
SIRT1	SRT1720	Aged ADH2 overexpression mice, cardiomyocyte function ↑; ROS↓	Zhang et al., 2014
	SRT3025	*ApoE* ^–/–^ mice, atherosclerosis↓; PCSK9↓, LDLR↑	Miranda et al., 2015
	E1231	*ApoE* ^–/–^ mice, atherosclerosis↓ LDL-C, TC, TG↓; ABCA1↑	Feng et al., 2018
	E6155	Type 2 diabetic mice, glucose homeostasis↑; p-AMPK↑, p-AKT↑	Liu et al., 2018a
Pan-sirtuin activation	NAD^+^	I/R rat model, infarction size↓; autophagy pathways↑	Zhang et al., 2020
	NMN	I/R mice model, infarction size↓; FOXO1 acetylation↓	Yamamoto et al., 2014
		Aging mice, arterial dysfunction, oxidative stress↓; NF-κB acetylation ↓	De Picciotto et al., 2016
		I/R mice model, infarction size↓; mitochondrial protein acetylation↓	Nadtochiy et al., 2018
		Aging mice, endothelial dysfunction↓, cognitive function↑; mitochondrial function↑	Tarantini et al., 2019
		Heart failure mice, LV dilation↓; mitochondrial protein acetylation↓	Lee et al., 2016
	NR	DIO mice, obesity↓, insulin, TC↓; energy expenditure↑	Canto et al., 2012
		TAC model mice, LVEF drop↓; glycolysis capacity↑	Diguet et al., 2018
		HFpEF mice, heart weight↓; mitochondrial function↑	Tong et al., 2021
		NAFLD mice, liver steatosis↓; mitochondrial function, β-oxidation↑	Gariani et al., 2016
		DIO mice, obesity unchanged; WAT inflammation↓, anti-oxidant gene↑	Shi et al., 2017
		DIO mice, obesity unchanged; WAT inflammation↑, glucose control↓	Shi et al., 2019
	NAM	HFpEF rats and mice, diastolic dysfunction improved; FA metabolism ↑, SERCA2a, titin deacetylation↑	Abdellatif et al., 2021
**Clinical**			
SIRT1	SRT2104	Type 2 diabetes; body weight↓	Noh et al., 2017
		Smoker; TC, LDL-C, TG↓	Venkatasubramanian et al., 2013
		Type 2 diabetes; body weight, TC↓	Baksi et al., 2014
Pan-sirtuin activation	NA	Metabolic syndromes; HDL-C↑, TG↓	Boden et al., 2011
	NMN	Prediabetic (overweight or obese); insulin signaling in skeletal muscle↑	Yoshino et al., 2021
	NR	Obese men; insulin resistance and liver lipid content unchanged	Dollerup et al., 2018
		Obese men without diabetes; glucose control unchanged	Dollerup et al., 2019
		Elderly; pro-inflammatory cytokines↓, glucose control unchanged	Elhassan et al., 2019
		Heart failure; pro-inflammatory cytokines↓	Zhou et al., 2020
	NAM	Higher NAM intake associated with lowered HFpEF risks, lowered blood pressure	Abdellatif et al., 2021

*ROS, reactive oxygen species; I/R, Ischemia-Reperfusion; NMN, nicotinamide mononucleotide; NR, nicotinamide riboside; NAM, nicotinamide; NA, nicotinic acid; LDL-C, low density lipoprotein cholesterol; TC, total cholesterol; TG, triglyceride; LV, left ventricle; DIO, diet-induced obesity; TAC, transverse aortic constriction; LVEF, left ventricle ejection fraction; HFpEF, heart failure with preserved ejection fraction; NAFLD, non-alcoholic fatty liver disease; FA, fatty acids; HDL-C, high density lipoprotein cholesterol.*

## Translating Experimental Findings to Patients

### Clinical Nicotinamide Adenine Diphosphate Booster Studies: Success and Pitfalls

Pharmacological approaches targeting sirtuins are also promising in preventing vascular disease in cardiometabolic patients. After resveratrol was shown to protect from metabolic diseases from energy-dense diets (Baur et al., 2006; Lagouge et al., 2006), numerous trials and animal experiments demonstrated that resveratrol can attenuate adipogenesis, inflammation, oxidative stress, and to rescue endothelial dysfunction, assessed by flow-mediated vasodilation. These effects on vascular function were mediated by the ability of a compound of inhibiting tumor necrosis factor (TNF) α-induced activation of NAD(P)H oxidase and preserving eNOS phosphorylation. Moreover, the lipid levels in the serum also decreased upon resveratrol treatment. The effects are thoroughly reviewed in another article (Dyck et al., 2019). The activating effect of resveratrol is direct on SIRT1 (Cao et al., 2015); however as discussed before, other sirtuins had their potential in providing the protection of CVDs, and thus a collective activation of all sirtuins using NAD^+^ boosters is of our interests.

The lipid-lowering properties of NA are known for decades: NA attracted clinical attention for its cholesterol lowering actions (Altschul et al., 1955), and became the first drug used to treat dyslipidemia. However, the clinical use of NA has been limited by the fact that it induces cutaneous flushing, which compromises compliance. This flushing derives from the activation of the G-protein-coupled receptor (GPCR) GPR109A (Benyo et al., 2005). Given the low levels of NA in blood, activation of GPR109A is possibly an effect from pharmacological dosing.

The AIM-HIGH (**A**therothrombosis **I**ntervention in **M**etabolic Syndrome with low HDL/**HIGH** Triglycerides) trial tested slow-release 1,500∼2,000 mg of NA in patients with cardiovascular diseases, most of whom took statins and antiplatelet or anticoagulant drugs at the starting point (Boden et al., 2011). Of interest, there was no prognostic benefit from the addition of niacin to statin therapy during a 36-month follow-up period, despite significant improvements in HDL cholesterol and triglyceride levels.

Although the effect on improving CVD incidents was not obvious, we want to point out that (1) the high dosing and extended release formulation of niacin used in the AIM-HIGH trial led to continuous activation of NAD^+^ pathways (sirtuins) and GPR109A (2) and that statins may have confounded data interpretation as they affect mitochondrial function (Bouitbir et al., 2012; Andreux et al., 2014). Since the lipid effects of NA are thought to be mediated *via* both the GPR109A and the NAD^+^-sirtuin axis (Houtkooper and Auwerx, 2012), an NAD^+^-centered approach not affecting GPCRs may be more advantageous.

NR is a compound that is first converted into NMN (Ratajczak et al., 2016), which can diffuse into mitochondria and nucleus, and then (by NMNATs) into NAD^+^. Thus, NR supplementation increases NAD^+^ pools of cytoplasm, mitochondria, and nucleus. Moreover, it does *not* activate the GPR109A, and NR is easily available via the internet or over-the-counter (Niagen^®^).

Following this concept, clinical studies using NR supplementation were recently reported. A group showed that supplementing NR 1,000 mg/day for 6 weeks had reasonable tolerability in middle-aged, lean, healthy subjects. NR supplement raised peripheral blood mononuclear cell (PBMC) NAAD and NAD^+^ concentrations and was accompanied with a trend of lower blood pressure (Martens et al., 2018). Another study with obese men (BMI > 30), 1,000 mg NR twice a day, increased the urinary metabolome of NAD^+^ in the NR group after 12 weeks. However, insulin resistance and liver lipid contents remained unaffected (Dollerup et al., 2018). Another intriguing study, though a very small sample size, demonstrated that NR treatment reduced the pro-inflammatory cytokine expressions from PBMC in heart failure patients (Zhou et al., 2020). This exhibits the potential of NR to suppress inflammation response and may be beneficial in heart failure patients.

Still, in several clinical studies, the results are not protective. Although Elhassan et al. (2019) recorded lower concentrations of pro-inflammatory cytokines in aged, healthy volunteers (70–80 years old), blood glucose control and insulin resistance were not affected by NR supplementation. Another study also showed no effects on glucose control after NR supplementation in obese men without diabetes (Dollerup et al., 2019).

Thus, NR supplementation so far appears relatively safe albeit the optimal supplement dosage, ideal target population and disease context require more sophisticated study design. Of note, all these studies were performed in small sample sizes and larger, randomized and blinded studies are warranted to test the putative clinical benefits of NR supplementation. We also summarize some of the clinical study results in Table 1.

### Lessons From Clinical Studies

The following parameters may account for the discrepancy between the NAD^+^ boosting benefits in mouse CVD models and their limited effects in human clinical trials.

#### Administration Route and Dosage

Crossing the species barrier when translating discoveries on model organisms to human clinical studies had always been challenging. NAD^+^ boosting using NMN had mostly relied on intraperitoneal injection, whereas NR and NAM given to mice had oral treatment. While many benefits have been identified with NMN supplementation, the route may dampen the convenience and accessibility of the compound. In addition, the high dosage used for mice treatment may not be applicable in humans (Canto et al., 2015). The 400∼500 mg/kg BW, though not directly translated to human supplementation, requires sophisticated calculation and evaluation to optimize the safe and reasonable dosage for human supplementation.

#### Precursor Metabolic Pathways

Second, the NAD^+^ precursor form and the pathways they convert into each other and NAD^+^. *In vivo* systems are complicated. Catabolic and anabolic pathways must be considered when an increase in a particular substance concentration should be achieved. For instance, a study showed that NAM supplementation did activate SIRT1, but was not accompanied by a raise in NAD^+^ level. It turned out that the hepatic catabolic enzymes of NAM are also raised, so supplemented NAM were catabolized (Mitchell et al., 2018). For NR and NMN supplementation, they differed at a phosphorylation step in chemical structure and NAD^+^ synthetic pathway. Thus, the enzymes of the phosphorylation interchange of NR and NMN, NRK1 and NRK2, are critical in the supplementation (Ratajczak et al., 2016). The regulation of NRK1 has been involved with liver steatosis and maintaining hepatic NAD^+^ levels (Fan et al., 2018). We should think of the regulation of these critical enzymes when examining the expected protective effects on cardiovascular diseases.

#### Inflammatory Responses in Humans

Third, although sirtuins are generally related to anti-inflammatory effects, several studies identified that expanding the NAD^+^ pool may be correlated with inflammatory responses. Treating human THP-1 cell with NA, NMN (Van Gool et al., 2009), or NR (Yang et al., 2019) increased intracellular NAD^+^ concentration and showed enhanced inflammation cytokine expression patterns. Immune cells are a critical part in many cardiovascular diseases, so we have to think carefully if human and rodent immune cells behave differently. Human SIRT6 is related to the expression of TNFα in the study (Van Gool et al., 2009), and of note, SIRT1 is not part of the NAD^+^ boosting-induced inflammation (Preyat et al., 2016; Yang et al., 2019). Thus, while we focus on SIRT1 -mediated effects when applying NAD^+^ boosters, we might overlook their effects of other sirtuins.

Therefore, when designing clinical trials to demonstrate the profits of NAD^+^ boosting, one should keep in mind that these differences may interfere with the study endpoints.

### Opportunities in the Clinical Arena

Despite the success and challenges in boosting the NAD^+^ pool with NAD^+^ precursors, they are never the only way to improve health through manipulation of NAD^+^ pathways and/or sirtuins. Numerous clinical and animal studies have been performed to imitate the beneficial effects of caloric restriction, showing improvement in metabolic diseases (Golbidi et al., 2017) and cardioprotection (Shinmura, 2013). In addition, a novel avenue showed the potential of antidiabetic drugs in stimulating the sirtuin pathway to provide protection in CVD.

#### Caloric Restriction

Caloric restriction, with the premise to fulfilling the nutrition requirement, can have beneficial impact on health. Although it remains a challenge to sustain such a diet in real life, it can be well controlled in experimental models. Caloric restriction benefits cardiovascular diseases through multiple facets. Loss of body weight, improvement in dyslipidemia, and suppression of immune response are the key benefits, and these benefits may be related to sirtuins and NAD^+^ pool regulation.

The most consistent benefit is probably aiding in losing body weight (Liu et al., 2016a; Yamamoto et al., 2016; Cohen et al., 2017; Miller et al., 2017; Waldman et al., 2018; Yu et al., 2018; Wei et al., 2020). The effect is seen in various experimental rodent models. The underlying mechanism is thought to be related to the raise of SIRT1 expression within the adipose tissue (Miller et al., 2017; Wei et al., 2020), and the elevation of NAD^+^ synthesizing enzyme NAMPT expression and to the fueling of NAD^+^ pool (Miller et al., 2017; Wei et al., 2020) to lose adipose tissue weight and/or reduce the lipid accumulation in adipocytes.

A second beneficial effect comprises an improved blood lipid profile. Caloric restriction treatment reduces total cholesterol (Cohen et al., 2017; Waldman et al., 2018), total triglycerides (Liu et al., 2016a; Waldman et al., 2018; Yu et al., 2018; Wei et al., 2020) in blood, and this poses a positive effect on cardiovascular diseases. Interestingly, it is accompanied with the raised expression of sirtuins in heart (Waldman et al., 2018; Yu et al., 2018) or aorta (Liu et al., 2016a) in rodent models, further contributing to the beneficial effects on reducing the risk of cardiovascular diseases.

Third, caloric restriction can decrease inflammation. As shown by reducing immune cell infiltration in heart and decreasing toll-like receptor (TLR) 2, TLR4, and TNFα in a diabetic mice model with cardiometabolic phenotypes (Cohen et al., 2017; Waldman et al., 2018). Caloric restriction is also found to reduce the systemic sterile inflammation in a diet-induced obesity mice model, where serum interleukin (IL)-6, and TNFα is lowered (Wei et al., 2020). These recent studies had shown that caloric restriction had their benefits which can be related to the elevation of NAD^+^ or raise in the expression or activities of sirtuins in the target tissue (adipose tissue for obesity, or aortae and heart for CVD). Although these discoveries are not totally unexpected for us, these data proved the benefits of caloric restriction more solidly and with more detail in molecular mechanisms.

#### Modulation of Sirtuin Signaling by Antidiabetic Drugs

##### SGLT2 Inhibitors

Much attention is raised in the SGLT2 inhibitor trials to treat type II diabetes and the accompanying protective effects on CVD. SGLT2, sodium-glucose cotransporter 2, accounts for the reuptake of 90% of filtrated glucose (Shubrook et al., 2015). By inhibiting the reuptake of glucose from the glomerular filtration back to the blood, these drugs enabled the body to excrete excess glucose and improved diabetes symptoms and complications. Surprisingly, these drugs provided potent protection in CVD, especially in heart failure. Although the exact molecular mechanism hasn’t been confirmed yet, effects may be—at least in part—related to caloric restriction and sirtuin activation.

To sum up these phase 3 studies, these inhibitors reduced death in cardiovascular diseases and hospitalization for heart failure (Zinman et al., 2015; Neal et al., 2017; McMurray et al., 2019; Wiviott et al., 2019; Cannon et al., 2020; Packer et al., 2020; Bhatt et al., 2021a,b). For those aimed at heart failure patients, they also demonstrated that the inhibitor works for both diabetic and non-diabetic patients (McMurray et al., 2019; Packer et al., 2020). This raised the question, whether these drugs work also for patients without diabetes, and whether they exert their effects through mechanisms independent of renal glucose excretion.

The activation mechanism may be the change of systemic energy balance. While being able to excrete glucose, it is conceivable that our body senses losing energy fuels and in turn stimulates biochemical pathways related to caloric restriction. Two major metabolic sensing signaling systems are AMPK and sirtuins, of which activity and expression are highly interconnected (Canto et al., 2009).

Studies showed that renal and hepatic SIRT1 expression is increased upon SGLT2 inhibition in mice (Umino et al., 2018; Swe et al., 2019). AMPK, a critical regulator of SIRT1, is also raised in the liver when treated with SGLT2 inhibitors, mimicking the molecular events in energy-deprived status (Swe et al., 2019). In addition, SGLT2 inhibition may increase adipocyte browning, potentially mediated by raising SIRT1 and PGC-1α expression in cultured mice adipocytes, and affect the systemic energy balance (Yang et al., 2020). This could further strengthen the energy-deprived status of the body and resemble caloric restriction even more. In addition to influencing whole body energy status, SGLT2 inhibitors can increase AMPK activity and affect glucose uptake and lipid oxidation *in vitro* (Hawley et al., 2016), which is a possible pathway to activate SIRT1. One SGLT2 inhibitor restored cardiac damage, raised expression of SIRT1, suppressed pro-inflammatory cytokine expression using a combined *in vivo*/*in vitro* approach (Ying et al., 2019). Thus, SGLT2 inhibitors may indirectly activate sirtuins.

Other than SIRT1, mitochondrial SIRT3 was demonstrated to participate in the protective effects of SGLT2 in experimental cardiomyopathy model, by forming a complex with TLR9 and Beclin1 (Wang et al., 2020), involving the innate immune system and autophagy pathways. The study identifies another member in sirtuin family to be involved in the protective effects of SGLT2 inhibitors.

##### Metformin

Beside SGLT2 inhibitors, another anti-diabetic drug has been shown to modulate sirtuins. Metformin, the first-line drug to treat hyperglycemia in patients with type II diabetes (Davies et al., 2018) may also affect sirtuin modulation. Metformin was found to modulate SIRT1-p66^*S*hc^ signaling in experimental models of diabetes (Paneni et al., 2013). Moreover, in PBMCs from patients with prediabetes, metformin significantly improved metabolic parameters and insulin sensitivity, increased SIRT1 gene/protein expression and SIRT1 promoter chromatin accessibility (De Kreutzenberg et al., 2015). The protection of metformin on cultured epithelial cells in the high glucose condition is also indispensable of SIRT1 (Arunachalam et al., 2014), supporting the notion that metformin conveys its benefits in part through regulating sirtuins.

Metformin was recently identified *in silico* and *in vitro* to directly bind to SIRT1 and serve as an agonist (Cuyas et al., 2018). Thus, like SGLT2 inhibitors, instead of eliciting other secondary responses, metformin may have the ability to directly interact with SIRT1 and to show benefits in CVD. This means that the potential benefits of metformin in CVD might work independent of its blood glucose-lowering effects. These findings could also explain the beneficial effects of metformin on cardiac remodeling and left ventricular mass in patients without diabetes (Mohan et al., 2019).

## Conclusion and Perspectives

Preclinical data demonstrate protective roles of sirtuins in cardiovascular and metabolic diseases by suppressing inflammation, improving lipid profiles, and scavenging oxidative stress, etc. Instead of activating specific sirtuins, boosting NAD^+^ levels may be more advantageous to harness protective effects of pan-sirtuin activation. While these approaches are promising in rodent models, some are not as positive. Clinical translation is even more challenging. Finally, a similar state of caloric restriction as induced with application of antidiabetic drugs may be mediated in part by sirtuin activation (Figure 1).

Taken together, additional mechanistic experimental studies and diligently planned clinical studies are needed to improve our understanding of the beneficial effects of sirtuin activation, NAD^+^ boosters and caloric restriction.

## Author Contributions

Y-JW and CM conceptualized the review article. FP and SS provided the critical review. All authors contributed to the article and approved the submitted version.

## Conflict of Interest

The authors declare that the research was conducted in the absence of any commercial or financial relationships that could be construed as a potential conflict of interest.

## Publisher’s Note

All claims expressed in this article are solely those of the authors and do not necessarily represent those of their affiliated organizations, or those of the publisher, the editors and the reviewers. Any product that may be evaluated in this article, or claim that may be made by its manufacturer, is not guaranteed or endorsed by the publisher.
